# Case report: Endoscopic Hemoclip treatment for massive hematemesis caused by a Dieulafoy’s lesion on duodenal papilla caused acute pancreatitis

**DOI:** 10.3389/fmed.2023.1108443

**Published:** 2023-03-02

**Authors:** Zhi-wei He, Hui Xu, Hua Shi, Yang-mei Wang, Han Zhong, Xiao-cong Liu

**Affiliations:** ^1^Ziyang First People’s Hosiptal, Ziyang, China; ^2^Chengdu Second People’s Hospital, Chengdu, China

**Keywords:** Dieulafoy’s lesion, duodenal papilla, hematemesis, endoscopic hemoclips, pancreatitis

## Abstract

**Background:**

Dieulafoy’s lesion is an uncommon cause of hemorrhage of the digestive tract. It often presents with urgent and massive bleeding usually leading to shock, even death. Dieulafoy’s lesions have been reported throughout the digestive tract but which occurred on duodenal papilla were particularly rare and presented challenges in the choice of hemostasis.

**Case presentation:**

A 66-year-old man with melena for 2 days was admitted. Gastrointestinal endoscopy revealed blood clots covering the duodenal papilla with oozing blood. During the procedure of trying to place a plastic stent into the duodenal papilla first, the hemorrhage began to present pulsating bleeding. The patient went into shock. With consent, two titanium clips were inserted to clamp the bleeding site to stop the bleeding. The patient complained of epigastric pain 14 h after the endoscopy. An abdominal CT scan showed signs of acute pancreatitis. Endoscopy was performed to remove the titanium clips and showed a vessel stump on the duodenal papilla. The patient was discharged from the hospital on the 14th day and followed for 6 months with no recurrence.

**Conclusion:**

This case was diagnosed with a Dieulafoy’s lesion on the duodenal papilla, which has rarely been reported. Hematemesis was stopped by clamping the vessel stump with titanium clips but caused acute pancreatitis. Reviewing the treatment, electrocoagulation might be a better choice, and life support treatment, including central vena catheterization and an adequate supply of blood products, should be prepared in advance to provide extra time for the stent placement or vascular intervention treatment.

## 1. Introduction

Dieulafoy’s lesion is a rare cause of hemorrhage of the digestive tract, in which a submucosal artery usually causes pressure necrosis of the overlying submucosa and mucosa and leads to hemorrhage. The bleeding caused by Dieulafoy’s lesion is usually urgent, causing shock and sometimes fatal. Dieulafoy’s lesions have been reported throughout the digestive tract but which occurred on duodenal papilla were particularly rare and presented challenges in the choice of hemostasis. Here, we present a case of massive hematemesis caused by a Dieulafoy’s located on duodenal papilla and the imperfect treatment.

## 2. Case presentation

A 66-year-old Asian man with melena was admitted to our hospital. The patient had had loose tarry stools two times a day (about 300 g each time) for 2 days without hematemesis before he was admitted. He did not complain about any abdominal pain or jaundice but felt dizzy and weak. He had coronary heart disease with a coronary stent placed 2 years previously and had been prescribed an ongoing therapy of aspirin 100 mg per day. The patient denied any positive family history.

**Figure 1:**
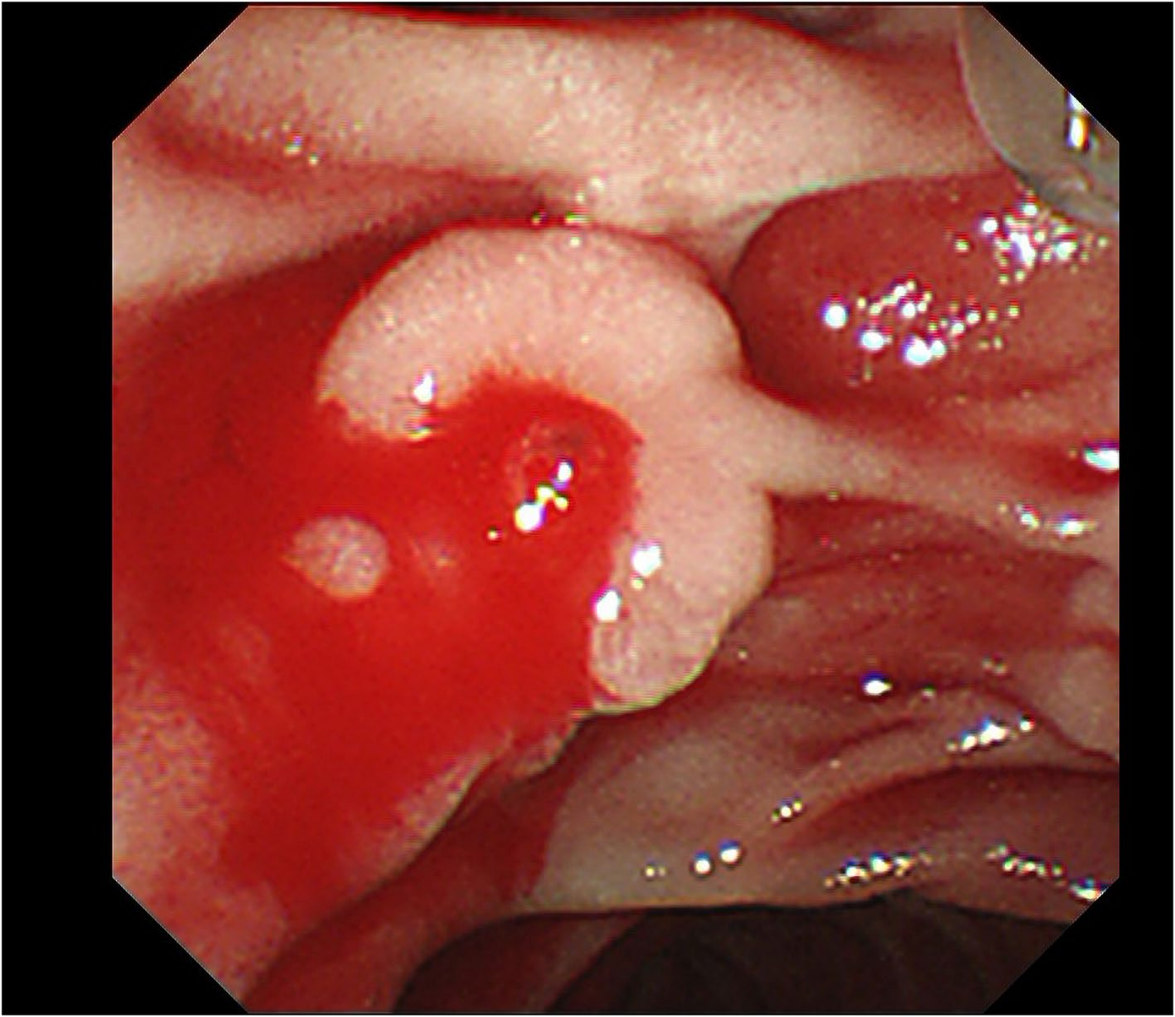
Pulsatile bleeding of the duodenal papilla after the clots were removed.

**Figure 2:**
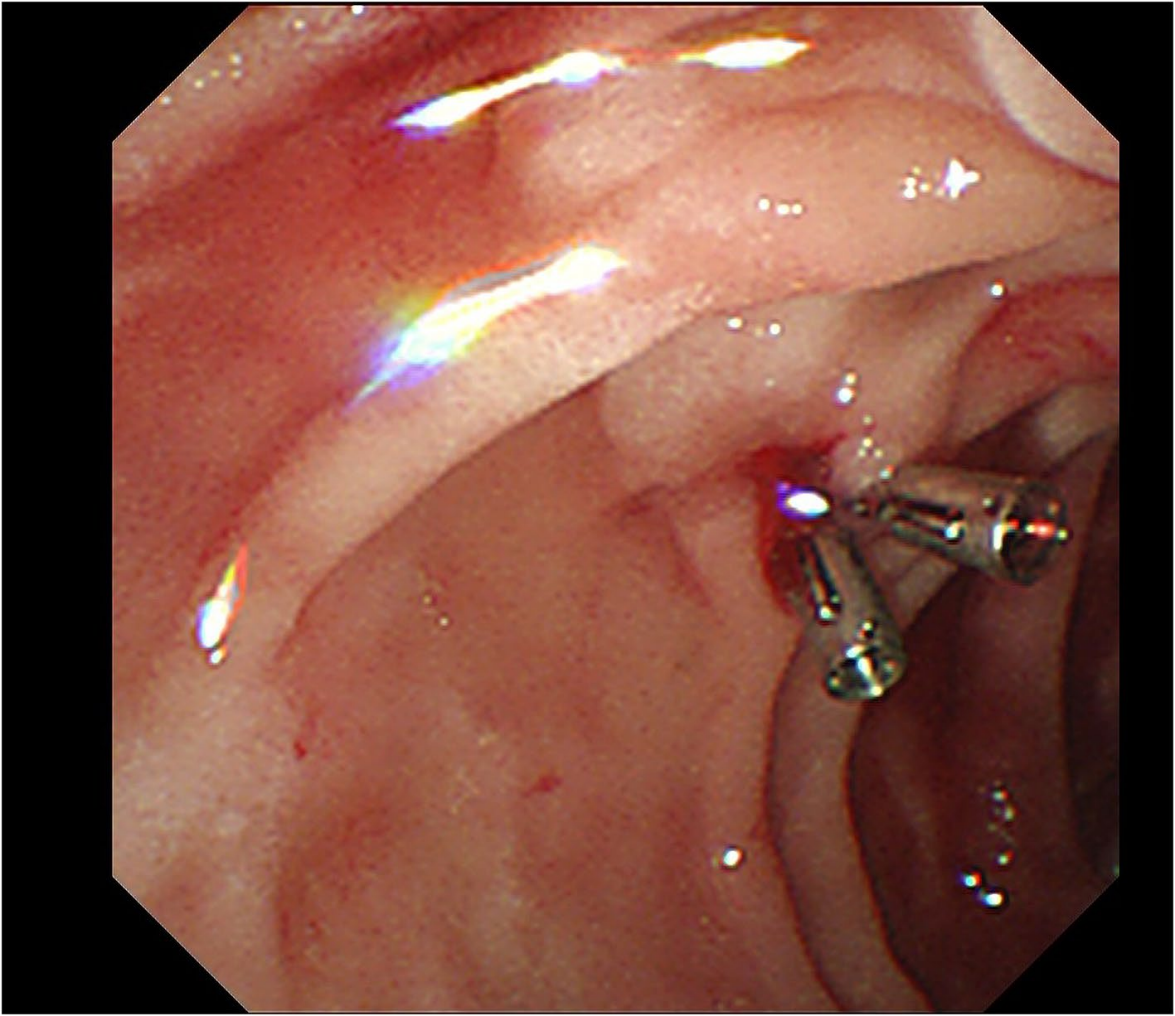
Two titanium clips were placed and the bleeding stopped.

**Figure 3:**
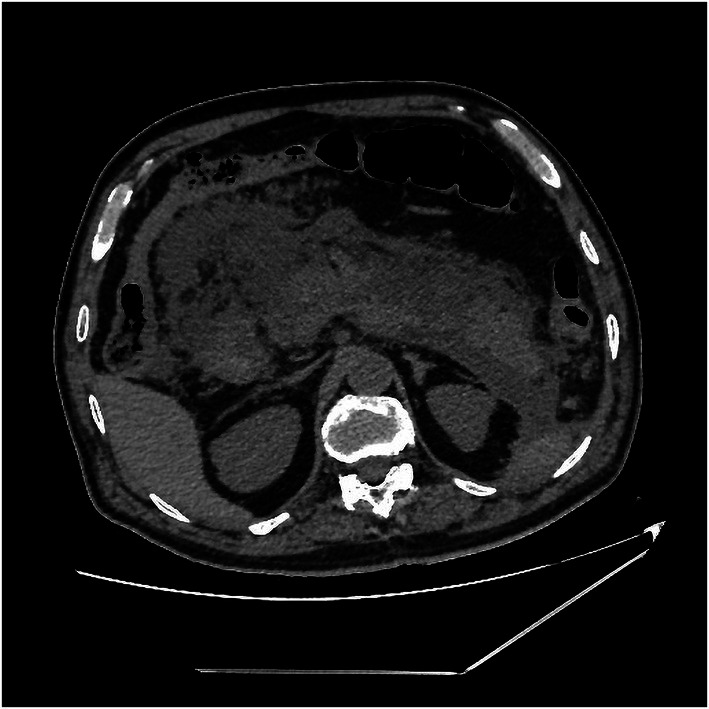
Abdominal CT showed diffuse enlargement of the pancreas and extensive peripancreatic exudation 14 h post endoscopy treatment.

**Figure 4:**
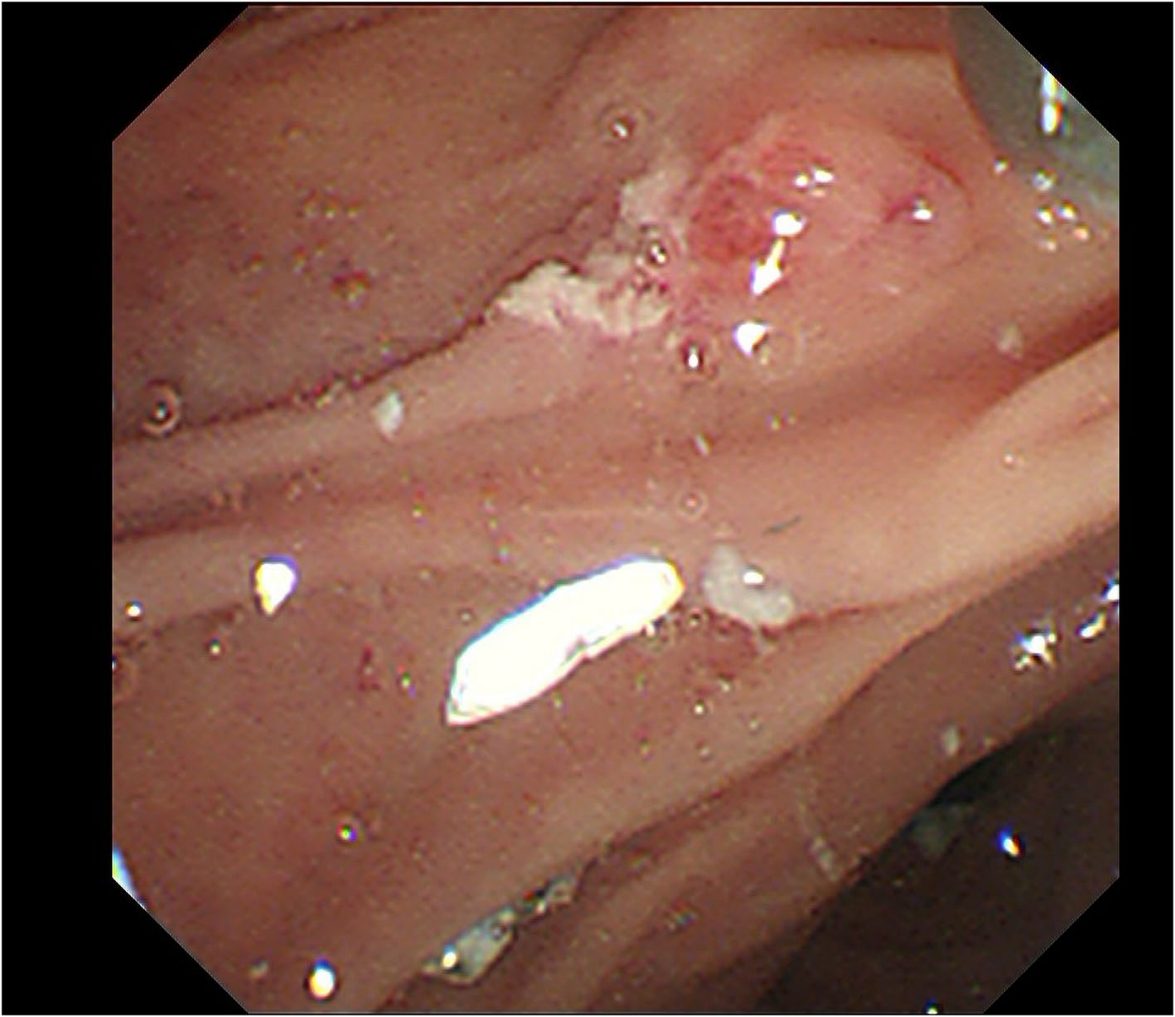
The Dieulafoy’s lesion was located at the 9 o ‘clock position of the duodenal papilla.

Upon admission, his temperature was 36.4°C, heart rate was 101 bpm, blood pressure was 112/74 mmHg, and respiration rate was 20 breaths/min. The patient was conscious and anemic looking, and bowel sounds were active. His abdominal color Doppler ultrasound was normal. Blood analysis revealed a total white blood cell count of 12.58 × 10^9^/L, red blood cell count of 2.69 × 10^12^/L, and hemoglobin of 82 g/l. The fecal occult blood test was (+). Liver and kidney biochemical and function tests were normal. The coagulation test revealed normal. After admission, the patient had nothing *per os* and received intravenous fluid resuscitation and proton pump inhibitors. An urgent upper gastrointestinal endoscopy was performed and revealed fresh red blood clots covering the duodenal papilla, with continued oozing blood from its basilar part, classified as Forrest Ib. The endoscopy was performed again with a duodenoscope for treatment. We planned to expose the opening of the duodenal papilla by removing the clots, sliding a catheter into the common bile duct and placing a plastic stent, and then clamping the hemorrhagic spot with titanium clips. During the procedure of locating the opening of the duodenal papilla after the clots were removed, the hemorrhage got worsened and began to present pulsating bleeding (Figure 1). Electrocardiography (ECG) monitor showed that the patient’s heart rate rose to 120–130 bpm and blood pressure dropped to 79/ 47 mmHg. The hemorrhage had to be stopped as soon as possible. We informed the patient’s son, who was the statutory agent of the patient, of the urgent situation and possible complications of clamping the bleeding spot without placing a bile duct stent previously, such as acute pancreatitis, obstructive jaundice, and acute obstructive suppurative cholangitis. With the consent, two titanium clips were inserted to clamp the bleeding site on the duodenal papilla. The 11 o’clock position and 1 o ‘clock position of the duodenal papilla were avoided as far as possible during the operation. The bleeding stopped immediately after the clips were placed (Figure 2). With further rapid intravenous fluid resuscitation and erythrocyte transfusion, the patient’s heart rate and blood pressure went normal. However, the patient complained of epigastric pain and abdominal distension 14 h later after the endoscopy. Abdominal computed tomography (CT) scan (no contrast was used) showed diffuse enlargement of the pancreas and extensive peripancreatic exudation (Figure 3). Other abnormal laboratory examination results were shown below: c-reactive protein 114.7 mg/l, serum amylase 2,368 U/l, aspartate aminotransferase 79 U/l, white blood cell count 21.16 × 10^9^/L, red blood cell count 3.38 × 10^12^/L, hemoglobin concentration 104.00 g/l, calcium 1.60 mmol/l, creatinine 133 μmol/l, lactate dehydrogenase 757 U/l, and α-hydroxybutyric acid 524 U/l. Acute pancreatitis was diagnosed. Endoscopy was performed immediately to remove the titanium clips. There was no re-bleeding after the clips were removed. The duodenal papilla was clearly observed, showing a vessel stump located at the 9 o ‘clock position (Figure 4). Then, the final diagnosis of the hemorrhage was massive upper gastrointestinal bleeding caused by a Dieulafoy’s lesion on the duodenal papilla. After further treatment of pancreatitis for 12 days, the related symptoms and indicators of pancreatitis were improved and no more bleeding occurred. The patient was discharged from the hospital on the 14th day and followed for 6 months with no recurrence.

## 3. Discussion

It is well known that Dieulafoy’s lesion is an uncommon cause of gastrointestinal hemorrhage. It is an abnormal submucosal artery, which runs a tortuous course (1). Dieulafoy’s lesions have been reported to be responsible for 1–5.8% of acute gastrointestinal bleeding (2, 3), but they typically present with severe, urgent, and massive bleeding, which usually causes hemodynamic instability, and even death. Sometimes, they are difficult to detect by endoscopy or surgery and have a high recurrence rate. Thus, Dieulafoy’s lesions could be life-threatening.

Dieulafoy’s lesion is characterized by an isolated protruding vessel, usually surrounded by a normal mucous membrane, or a small round or oval superficial ulcer (4, 5). The vessel stump is usually between 1 and 3 mm in diameter (6). Bleeding caused by Dieulafoy’s lesion may be spurting or oozing, or the lesion may be covered by a blood clot. Dieulafoy’s lesions can be distributed in all segments of the digestive tract, including 8% in the esophagus, 71% in the stomach, 15% in the duodenum, 1% in the intestine, 2% in the colon, 2% in the rectum, and 1% in gastric anastomosis (2). We previously reported a Dieulafoy’s lesion that occurred inside a duodenum diverticulum (7), which is rare (only five cases reported) in total. However, Dieulafoy’s lesions, which occurred on duodenal papilla, were particularly rare, and only two cases were reported previously (8, 9). In the current case, the vessel stump of the lesion is located at the 9 o ‘clock position of Duodenal papilla, close to the opening, in the absence of an ulcer, confirming the diagnosis of a Dieulafoy’s lesion. Commonly used endoscopic hemostatic procedures for Dieulafoy’s lesions include thermocoagulation, electrocoagulation, sclerotherapy, banding, local epinephrine injection, and hemoclips (6, 10). In the previously reported two cases, argon plasma coagulation, epinephrine injection, and bipolar cautery were used to stop the bleeding. The reasons we chose to use hemoclips include that it was a routinely used hemostatic method by us during the ERCP procedure, and it was easier to adjust the clips to avoid the 11 and 1 positions, which were the common openings of the bile duct and pancreatic duct, respectively. We tried to place a plastic stent into the duodenal papilla before the hemoclip treatment to prevent the occurrence of pancreatitis, but the bleeding got worse during finding the opening of duodenal papilla and the patient showed the signs of shock. There was no time left to continue the procedure of intubation and stent placement or transfer the patient to the interventional room for vascular intervention. After the necessary informed consent, we clamped the bleeding by hemoclips and tried to avoid the opening positions of the bile duct and pancreatic duct. Unfortunately, the patient got acute pancreatitis after the hemoclip treatment. Reviewing the treatment, electrocoagulation may be a better choice in that emergency situation, though there would still be a risk of acute pancreatitis due to duodenal papilla edema caused by electrocoagulation. Furthermore, life support treatment, including central vena catheterization, adequate supply of erythrocyte suspension, and plasma, should be prepared in advance, for the possibility of providing extra time for the stent placement or vascular intervention treatment.

## Data availability statement

The original contributions presented in the study are included in the article/supplementary material, further inquiries can be directed to the corresponding author.

## Ethics statement

Written informed consent was obtained from the individual(s) for the publication of any potentially identifiable images or data included in this article.

## Author contributions

Z-wH: endoscopy operation. HX: endoscopy operation guidance. HS: supervising physician of the patient. Y-mW: literature search. HZ: endoscopy operation assistant. X-cL: acquired all data and wrote the manuscript. All authors contributed to the article and approved the submitted version.

## Conflict of interest

The authors declare that the research was conducted in the absence of any commercial or financial relationships that could be construed as a potential conflict of interest.

## Publisher’s note

All claims expressed in this article are solely those of the authors and do not necessarily represent those of their affiliated organizations, or those of the publisher, the editors and the reviewers. Any product that may be evaluated in this article, or claim that may be made by its manufacturer, is not guaranteed or endorsed by the publisher.
